# Therapeutic effect of GLP-1 engineered strain on mice model of Alzheimer’s disease and Parkinson’s disease

**DOI:** 10.1186/s13568-020-01014-6

**Published:** 2020-04-24

**Authors:** Xin Fang, Xiaoting Zhou, Yuqing Miao, Yiwen Han, Jing Wei, Tingtao Chen

**Affiliations:** 1 Department of Neurology, The First Affiliated Hospital of Nanchang University, Nanchang, China; 2 National Engineering Research Center for Bioengineering Drugs and the Technologies, Institute of Translational Medicine, Nanchang University, 1299 Xuefu Road, Honggu District, Nanchang, 330031 Jiangxi People’s Republic of China

**Keywords:** Alzheimer’s disease (AD), Parkinson’s disease (PD), Engineered bacteria, High-throughput sequencing, Neuroinflammation, Glucagon-like peptide-1 (GLP-1), MG1363-pMG36e-GLP-1

## Abstract

Alzheimer’s disease (AD) and Parkinson’s disease (PD) are neurodegenerative diseases (NDD) characterized by progressive degeneration of the central nervous system, and few medications are available to halt the progression of AD and PD. In the present study, an engineered strain MG136-pMG36e-GLP-1 was used to evaluate its neuroprotective effect on AD and PD mice, via the probiotics effects of *Lactococcus lactis* MG1363 and the constantly produced Glucagon-like peptide-1 (GLP-1) by the engineered strain. Our results indicated that oral administration of MG136-pMG36e-GLP-1 significantly reduced lipopolysaccharide (LPS)-induced memory impairment and 1-methyl-4-phenyl-1,2,3,6-tetrahydropyridine (MPTP)-induced motor dysfunction through the toll-like receptor4 (TLR4)/nuclear factor-kappa B (NFκB) and protein kinase B (AKT)/Glycogen synthase kinase-3β (GSK3β) signaling pathway. High-throughput sequencing results showed that MG1363-pMG36e-GLP-1 reduced the abundance of the pathogens *Enterococcus*, *Proteus*, and increased the abundance of the probiotics *Akkermansia muciniphila*. These results suggest that the engineered strain may be a new intervention for treating AD and PD by reducing the occurrence of neuroinflammation.

## Key points

- An engineered strain MG1363-pMG36e-GLP-1 is constructed which continuously express GLP-1, and overcome the problem of the short half-life of GLP-1.
- MG1363-pMG36e-GLP-1 relieves memory disorders in Alzheimer’s disease (AD) mice and dyskinesias in Parkinson’s disease (PD) mice, and reduces their neuroinflammation.
- MG1363-pMG36e-GLP-1 can be developed as oral GLP-1 drugs with few side effects, low cost, and no need for repeated injections.

## Introduction

Neurodegenerative diseases (NDD) are a group of diseases caused by chronic progressive degeneration of the central nervous system, e.g. Alzheimer’s disease (AD), Amyotrophic lateral sclerosis (ALS) and Parkinson’s disease (PD). Although NDD have various lesions with mostly unclear pathogenesis, some common features of progressive degeneration and necrosis of neurons of NDD have been identified, and it is key to develop drugs to treat NDD via overcoming the limited understanding of their etiology and mechanism (Gitler et al. 2017).

As the most common NDD, AD and PD greatly affect the quality of life. The pathological features of AD are neurofibrillary tangles caused by tau hyperphosphorylation and deposition of amyloid β (Aβ) in the cortex, hippocampus and amygdala. The main clinical manifestations of AD are progressive memory loss, cognitive dysfunction and language impairment (Du et al. 2018). PD is pathologically characterized by the death of dopaminergic neurons in the substantia nigra pars compacta (SNpc) and Lewy bodies with α-synuclein (α-syn) as the main component in the residual cytoplasm of neurons. The clinical manifestations for PD patients include quiescent tremor, bradykinesia, muscular rigidity, postural balance disorder, and non-motor symptoms caused by autonomic dysfunction (Sveinbjornsdottir 2016). Although the pathological features and clinical manifestations of AD and PD are different, they are all caused by misfolding of endogenous proteins. The deposition of Aβ and α-syn activates microglia in the brain and triggers neuroinflammation, which leads to memory and cognitive impairment in AD patients and motor and non-motor symptoms in PD patients (Rogers et al. 2007). With the advent of an aging population, more and more people are suffering from AD and PD, but only a few drugs are available for the two diseases mentioned above, and they can only relieve symptoms but fail to halt the progression of the diseases (Du et al. 2018; Grimes et al. 2019). Therefore, developing drugs targeting common contributors of AD and PD may be a wise strategy.

There are approximately 37 trillion microorganisms in the human body. They live on and in the body, 70% of which are in the intestine (Spielman et al. 2018). Normal intestinal microbes have a steady state balance with immune, endocrine, digestive and other physiological functions. These microbes have the ability to protect the host from pathogen invasion, promote host digestion and absorption, regulate drug metabolism, innate immunity and acquired immune system (Clemente et al. 2012). Braak first proposed the idea that PD may originate in the gastrointestinal tract (Del Tredici and Braak 2008). Subsequent research confirmed this conjecture and showed that intestinal microbes could regulate neurophysiological functions through the neurological, endocrinological and immunological pathways (Sampson et al. 2016). Intestinal microbes are closely related to neurological diseases such as AD, PD and ALS. In the stools of patients with AD, the abundance of pro-inflammatory bacteria (*Escherichia/Shigella*) increases and the abundance of anti-inflammatory bacteria (*Eubacterium rectale*) and *Bacteroides fragilis* decreases (Mancuso and Santangelo 2018). Similarly, the abundance of *Escherichia*-*Shigella*, *Proteus*, and *Enterococcus* was increased, and the abundance of *Blautia* and *Ruminococcus* was decreased in the faecal samples of PD patients (Sampson et al. 2016). AD and PD are accompanied by impaired intestinal barrier (Forsyth et al. 2011), lipopolysaccharide (LPS) and pro-inflammatory factors produced by pathogens can enter the human body through the damaged intestinal barrier, destroy the integrity of the blood–brain barrier (BBB) and increase the brain’s uptake of Aβ and α-syn, thus activate microglia by LPS/toll-like receptor4 (TLR4)/nuclear factor-kappa B (NFκB) pathway to induce the immune response, which ultimately leads to the loss of neurons (Dutta et al. 2008; Lund et al. 2011).

Glucagon-like peptide-1 (GLP-1) is an endogenous hormone secreted by ileal endocrine cells, which promotes insulin secretion in a hyperglycemic environment (Athauda and Foltynie 2016). There are studies indicate that GLP-1 has a neuroprotective effect in the central nervous system (CNS), which affects the proliferation and apoptosis of neural cells, improves learning, memory and motor dysfunction, reduces the deposition of Aβ plaques in the brain, decreases the loss of dopaminergic neurons and promotes nerve regeneration (Kim et al. 2017). Currently, GLP-1 drugs have been used in AD and PD patients in clinical trials and have shown efficacy (Aviles-Olmos et al. 2013; Gejl et al. 2016). However, when GLP-1 enters the body, it is recognized and degraded by dipeptidyl peptidase IV (DPP-IV), and its plasma half-life is less than 2 min. In our previous work, an engineered *Lactococcus lactis* strain MG1363-pMG36e-GLP-1 was constructed, which could continuously express GLP-1. It shows a neuroprotective effect on LPS-induced AD mice and 1-methyl-4-phenyl-1,2,3,6-tetrahydropyridine (MPTP)—induced PD mice by reducing the occurrence of neuroinflammation, which significantly alleviates LPS-induced spatial learning, memory disorders and MPTP-induced dyskinesia (Chen et al. 2018; Fang et al. 2019). However, why it has a sound effect both on AD and PD has not been fully elucidated. In the present study, the LPS-induced AD mice and MPTP-induced PD mice were developed to explore the effect of MG1363-pMG36e-GLP-1 on AD and PD. Behavioral assessment, immunohistochemistry, immunofluorescence, high-throughput 16S rDNA gene amplicon analysis are applied to find the common mechanisms of MG1363-pMG36e-GLP-1 on NDD, providing useful data on the development of neuroprotective drugs.

## Materials and methods

### Animals and administration

**Fig. 1:**
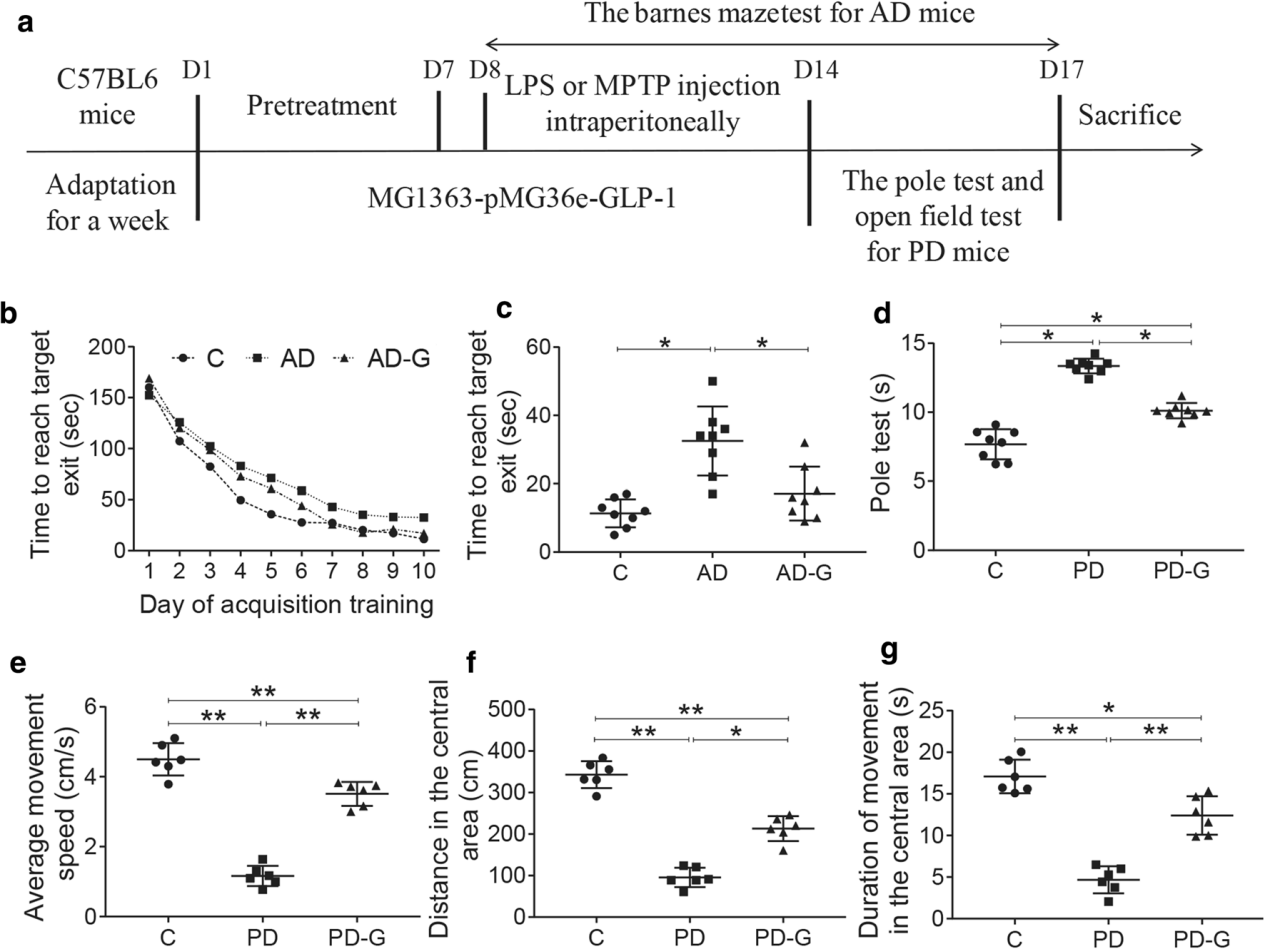
MG1363-pMG36e-GLP-1 improved cognitive ability in AD mice and motor function in PD mice. **a** The time schedule to explain the design of the whole experimental design. **b** The escape latency of mice in the Barnes maze test during the acquisition phase (1–10 days). **c** The escape latency of mice in the Barnes maze test on the last day. MG1363-pMG36e-GLP-1 had greatly reduced the escape latency. **d** MG1363-pMG36e-GLP-1 improved the bradykinesia mice induced by MPTP (pole test). **e** MG1363-pMG36e-GLP-1 increased the distance in the central area moved for PD mice (open-field test). **f** MG1363-pMG36e-GLP-1 increased the average movement speed for PD mice (open-field test). **g** MG1363-pMG36e-GLP-1 increased the duration of movement in the central area for PD mice (open-field test). C, control group (n = 8); AD, LPS group (n = 5); AD-G, LPS + 10^9^ CFU MG1363-pMG36e-GLP-1 group (n = 5); PD, MPTP group (n = 8); PD-G, MPTP + 10^9^ CFU MG1363-pMG36e-GLP-1 group (n = 8). Data are presented as mean ± SD. *p < 0.05, **p < 0.01

Male C57BL/6 mice weighing 25–30 g were provided by Hunan SJA laboratory animals (Changsha, Hunan, China). The mice were kept in an environment with a 12/12 dark cycle, a humidity of 55 ± 5%, and a temperature of 23 ± 2 °C. Water and food can be freely taken by mice. All experiments are performed from 9:00 a.m. to 12:00 noon to avoid errors caused by experimental time. All mice were divided into 5 groups randomly (n = 12 per group). The five groups were: (1) a control group treated with saline (C), (2) a group treated by LPS injection intraperitoneally for 7 days for 0.25 mg/kg body weight per day (AD), (3) the AD mice pretreated with MG1363-pMG36e-GLP-1 (AD-G), (4) a group treated by MPTP injection intraperitoneally for 7 days for 20 mg/kg body weight per day (PD), (5) The PD mice pretreated with MG1363-pMG36e-GLP-1 (PD-G). MG1363-pMG36e-GLP-1 strains were administered in drinking water. For the pretreatment group (AD-G and PD-G), 10^9^ CFU MG1363-pMG36e-GLP-1 strain was administered daily for 7 days before the injection of LPS and MPTP per day, and was administered daily for a total of another 2 weeks. When the behavior tests were finished, the mice were sacrificed after anesthesia by an intraperitoneal injection of ketamine (Rotex, Trittau, Germany). All samples (feces and brain tissues) were kept for further use at a − 80 °C (Fig. 1a).

### Behavioral assessment

#### Barnes maze test

The barnes maze is a model for detecting spatial memory and learning abilities of animals. On the first day of the experiment, mice were placed in the avoidance box (target box) from the target hole for 3 min, and then placed in the start box in the center of the maze for 3 min. Prior to behavioral testing, mice had a 10-day acquisition phase. After the scheduled time, the mice were placed in the middle of the maze and moved freely during the test. The experimenter monitors the behavior of the mice through the camera. When the animal put all four paws into the target box, it was counted as an escape and the mice were allowed to stay in the box for 30 s. The animal was guided into the target box and left for 30 s, if it did not find the target box within 3 min. The observation of the time when the mouse head entered the target hole and the number of times the mouse entered the wrong hole was operated by placing the mice in a maze for 3 min on the last day. Each mouse was cleaned of excretion after the test was completed and wiped with alcohol to remove the odor.

#### Pole test

The pole test was used to assess the Bradykinesia in PD mice. Mice were placed head down on the top of a metal rod wrapped in gauze. The time it took for the mice to travel from the top down to the hind legs to the bottom of the cage was recorded. Each mouse was tested 3 times with a 15 min interval between each test.

#### Open field test

The observation of the autonomous behavior, inquiry behavior and tension of experimental animals in new environments were supported by the application of the open field experiment. The open field test consisted of a square plastic box (50 cm * 50 cm * 40 cm) divided into 25 square grids of equal size. During the experiment, the mouse was put in from the corner of the field instrument, and the process of exploring the mouse in the device for 5 min was recorded with a camera. After each mouse experiment, the device was wiped thoroughly with alcohol.

### Sample collection and preservation

For immunohistochemistry, 5 mice in each group were anaesthetized with ketamine after the behavioral test. Mice were perfused with 45 mL saline into the left cardiac apex and then with 180 mL of PBS containing 4% paraformaldehyde. Their brains were fixed in 4% (v/v) paraformaldehyde after immediate obtainment. Other mice were directly taken the brain after anesthesia. All fecal samples were collected and kept for further use at a − 80 °C.

### Immunohistochemistry

The fixed brain tissue was removed from 4% (v/v) paraformaldehyde and embedded in paraffin. Samples were sliced into sections of 5 μm thick and rehydrated by xylene (Sigma Cat #247642) followed by a series of declining concentration of ethanol for 5–6 min,then each slide was washed by PBS with 6 min for 4 times. Non-specific binding was avoided by blocking the sections into 3% horse serum (HyClone SH30074.03). Then the sections were incubated with mouse anti-toll-like receptor 4 (TLR4; 1:500; Santa Cruz Biotechnology, Cat# sc-293072), rabbit anti-NFκB/P65 (NF-κB; 1:800; Cell Signaling Technology, Cat# 8242), rabbit anti-phosphorylated-NFκB/P65 (p-NF-kB; 1:1000; Abcam, Cat# ab86299), mouse anti-phosphorylated-I kappa B alpha (p-IκB-α; 1:500; Santa Cruz Biotechnology, Cat# sc-8404), mouse anti-I kappa B alpha (IκB-α; 1:500; Santa Cruz Biotechnology, Cat# sc-1643), rabbit anti-Phospho-GSK3β (p-GSK3β; 1:100; Abcam, Cat# ab75745), mouse Anti-GSK3 beta (GSK3β; 1:500; Santa Cruz Biotechnology, Cat# sc-7291), mouse anti-AKT-Phospho-S473 (p-AKT; 1:400; Proteintech, Cat# 66444-1-Ig), rabbit Anti-AKT (AKT; 1:1000; Proteintech, Cat# 10176-2-AP), rabbit Anti-Beta-Catenin (β-catenin; 1:2000; Proteintech, Cat# 51067-2-AP) primary antibody at 4 °C overnight. Primary antibodies were detected by using proper secondary antibodies, e.g. goat anti-rabbit secondary antibody (1:200; Servicebio, Cat# GB23303), or goat anti-mouse secondary antibody (1:200; Servicebio, Cat# GB23301).

### Immunofluorescence

The fixed brain tissue was removed from 4% (v/v) paraformaldehyde and embedded with paraffin. Samples were sliced into sections of 5 μm thick, and the sections were mounted on glass slides. After blocking with serum for 30 min, the sections were subjected to rabbit anti-beta amyloid (Aβ; 1:100; Cell Signaling Technology, Cat# 8243), rabbit anti-TH (TH; 1:500; Proteintech, Cat# 25859-1-AP) primary antibody incubate at 4 °C overnight. Finally, the sections were washed and incubated with secondary antibody Cy3 goat anti-rabbit antibody (1: 300; Servicebio, catalog number GB21303) at room temperature for 50 min.

### Microbiota analysis

For microbiota analysis, faecal samples of groups C (n = 8), AD (n = 5), AD-G (n = 5), PD (n = 8) and PD-G (n = 8) were collected. Total fecal DNA was extracted using a Genomic DNA kit (Qiagen, Cat # 51804) and the extraction was supported by the method of bead beating. The concentration of genomic DNA in each fecal sample was quantified using a NanoDrop spectrophotometer. Primers (515F, 5′-GTGCCAGCMGCCGCGGTAA-3′; 806R,5′-GGACTACVSGGGTATCTAAT-3′) targeting the V4 variable region of microbial small subunit (SSU or 16S) ribosomal RNA (rRNA) genes were used for PCR, the products of PCR were sequenced with an IlluminaHiSeq2000 platform (GenBank accession number PRJNA577377).

Using QIIME software, the UCLUST sequence alignment tool is invoked to combine and divide the above sequences according to 97% sequence similarity. The most abundant sequence in each operational taxonomic unit (OTU) was selected as the representative sequence. After that, Qiime software (version 1.9.1) was used to analyse α diversity and β diversity. Commonly, four measurement indexes are used to analyse α diversity of the flora. Chao1 index and ACE index mainly reflect the richness of the community, and Shannon index and Simpson index consider the uniformity of the community. The main objective of β diversity analysis is to investigate the similarity of community structure among different samples. To decompose the structure of community data and observe the differences among the ordering samples, Principal Component Analysis (PCA), Multidimensional Scaling (MDS) and Clustering Analysis were used. PCA analysis of genus-level community composition and Nonmetric Multidimensional Scaling (NMDS) analysis of Unweighted and Weighted UniFrac distance matrices were performed using R software. UPGMA clustering analysis was performed on Unweighted and Weighted UniFrac distance matrices using QIIME software, and visualized using R software.

### Data analysis

Prism software version 7.0 (GraphPad Software, San Diego, CA, USA) was used for data analysis. Statistical analysis was conducted by one-way analysis of variance (ANOVA) followed by Tukey’s multiple comparison tests. Data are presented as mean ± SD (Standard Deviation), and p < 0.05 was set as the threshold for significance.

## Results

### MG1363-pMG36e-GLP-1 improved cognitive ability in AD mice and motor function in PD mice

The Barnes maze test was used to test the effect of MG1363-pMG36e-GLP-1 engineered strain on the cognitive ability of AD mice induced by LPS. Compared with the C group, mice in AD group exhibited cognitive impairment which notably prolonged the time for mice to find the platform (p < 0.05). However, taking of MG1363-pMG36e-GLP-1 had greatly reduced the escape latency (p < 0.05; Fig. 1b). From the 7th day, the latency period of searching for right foramen in AD-G group was gradually shortened, which was similar with that in C group. On the last day, the latency time of AD-G group was shorter than that in AD group (17.13 s vs 31.25 s) (p < 0.05; Fig. 1c).

The total descent time was measured in pole test for evaluation of bradykinesia. PD mice showed longer pole descent time (7.67 s vs 13.35 s) contrast to the normal control group (p < 0.001). However, treatment with MG1363-pMG36e-GLP-1 showed improved performance in pole descent test (10.11 s) (p < 0.001, Fig. 1d). In the open field assessment, PD mice had reduced the average movement speed (1.16 s vs. 4.5 s), the distance in the central area (95.73 cm vs. 343.1 cm) and the duration of movement in the central area (4.67 s vs. 17.1 s) versus the control group (p < 0.001; Fig. 1e–g), and treatment with MG1363-pMG36e-GLP-1 had significantly improved the behavioral performance compared with mice in PD group (p < 0.001; Fig. 1e–g).

### MG1363-pMG36e-GLP-1 reversed the pathological changes induced by LPS and MPTP

**Fig. 2:**
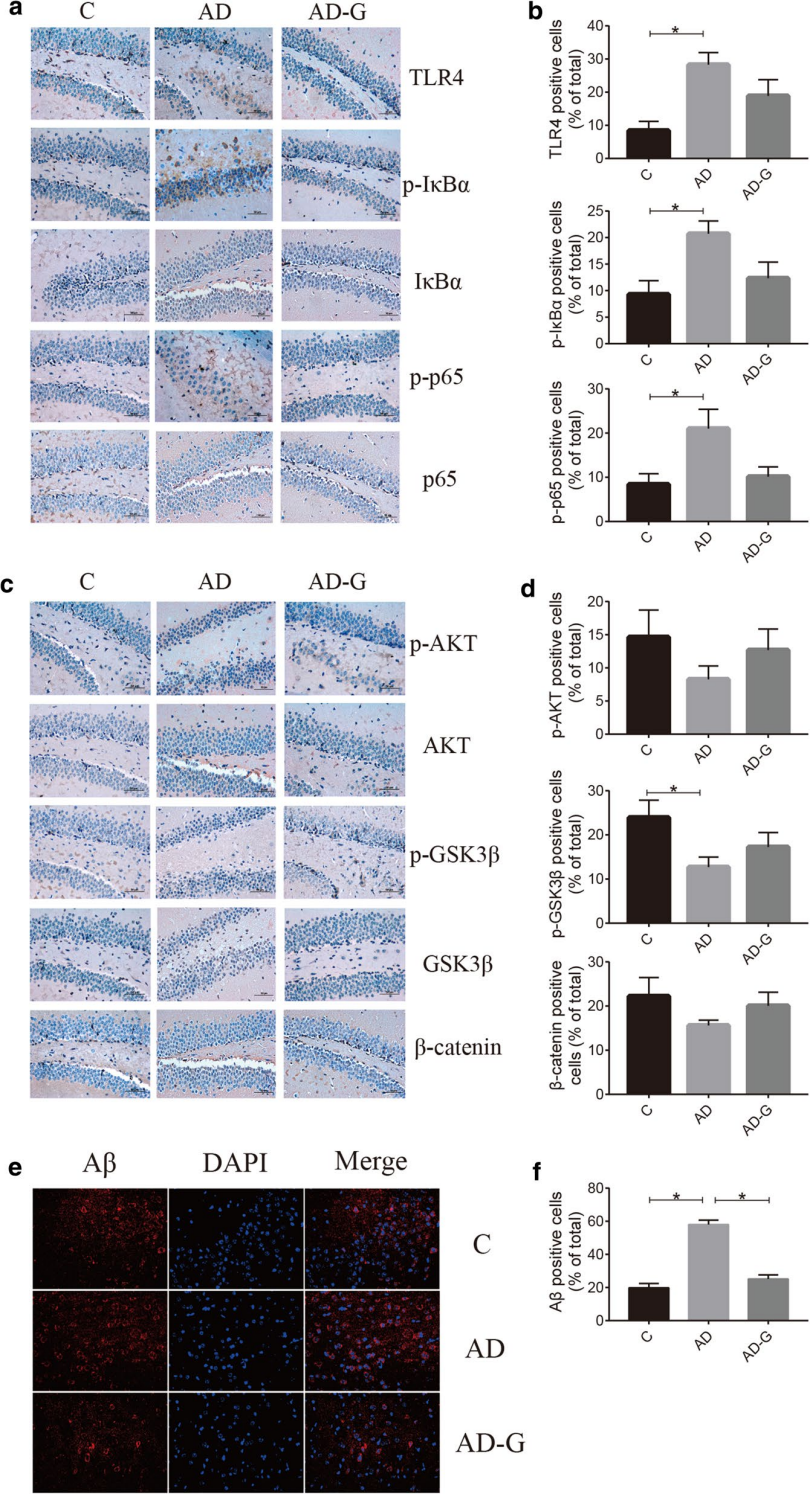
MG1363-pMG36e-GLP-1 reversed the pathological changes induced by LPS. **a** MG1363-pMG36e-GLP-1 reduced the expression of TLR4, p-IκBαand p-p65 on mouse brain induced by LPS (IHC staining of hippocampus). **b** MG1363-pMG36e-GLP-1 decreased the expression of p-AKT/AKT, p-GSK3β/GSK3βand β-catenin on mouse brain induced by LPS (IHC staining of hippocampus). **c** MG1363-pMG36e-GLP-1 alleviated the Aβdeposition on mouse brain induced by LPS (IF staining of hippocampus). C, control group (n = 8); AD, LPS group (n = 5); AD-G, LPS + 10^9^ CFU MG1363-pMG36e-GLP-1 group (n = 5); PD, MPTP group (n = 8); PD-G, MPTP + 10^9^ CFU MG1363-pMG36e-GLP-1 group (n = 8). Data are presented as mean ± SD. *p < 0.05

**Fig. 3:**
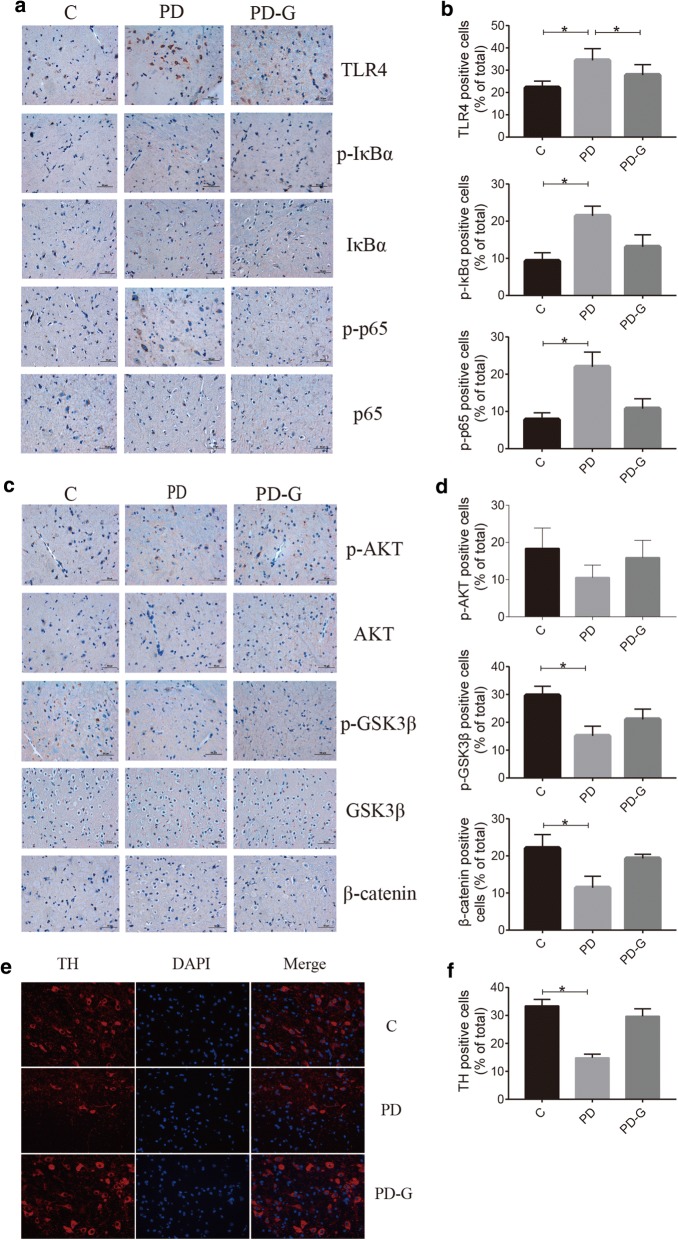
MG1363-pMG36e-GLP-1 reversed the pathological changes induced by MPTP. **a**, **b** MG1363-pMG36e-GLP-1 reduced the expression of TLR-4, p-IκBαand p-p65 on mouse brain induced by MPTP (IHC staining of substantia nigra). **c**, **d** MG1363-pMG36e-GLP-1 decreased the expression of p-AKT/AKT, p-GSK3β/GSK3β and β-catenin on mouse brain induced by MPTP (IHC staining of substantia nigra). **e**, **f** MG1363-pMG36e-GLP-1 alleviated the reduction of dopamine neurons on mouse brain induced by MPTP (IF staining of substantia nigra). C, control group (n = 8); AD, LPS group (n = 5); AD-G, LPS + 10^9^ CFU MG1363-pMG36e-GLP-1 group (n = 5); PD, MPTP group (n = 8); PD-G, MPTP + 10^9^ CFU MG1363-pMG36e-GLP-1 group (n = 8). Data are presented as mean ± SD. *p < 0.05

LPS and MPTP could enhance the release of proinflammatory cytokines, which can contribute to neuroinflammation and lead to the apoptosis of brain cells. Therefore, immunohistochemistry (IHC) technology was used to detect the expression of inflammatory factors and apoptotic factors in brain tissues, and immunofluorescence (IF) technology was used to observe the amyloidogenesis (Aβ) in AD mice and the TH-positive neurons in PD mice. As shown in Figs. 2 and 3, intraperitoneal injection of LPS or MPTP had obviously increased the positive cells for TLR4, p-IκBαand p-p65, while decreased the nmbers of positive cells for p-AKT, p-GSK3βand β-catenin in brain tissue contrast to the C group, while treatment with MG1363-pMG36e-GLP-1 had reversed this trend (Figs. 2a–d and 3a–d) (p < 0.05). The injection of LPS had obviously increased the Aβ accumulation, while treatment with MG1363-pMG36e-GLP-1 had reversed this trend (Fig. 2e, f) (p < 0.05). TH-positive dopaminergic neurons were significantly lost in MPTP mice compared with normal control mice. Treatment with MG1363-pMG36e-GLP-1 partially rescues the decrease of TH-positive neurons (Fig. 3e, f) (p < 0.05).

### Dysbiosis of the gut microbiota in AD and PD mice was reduced by MG1363-pMG36e-GLP-1

**Fig. 4:**
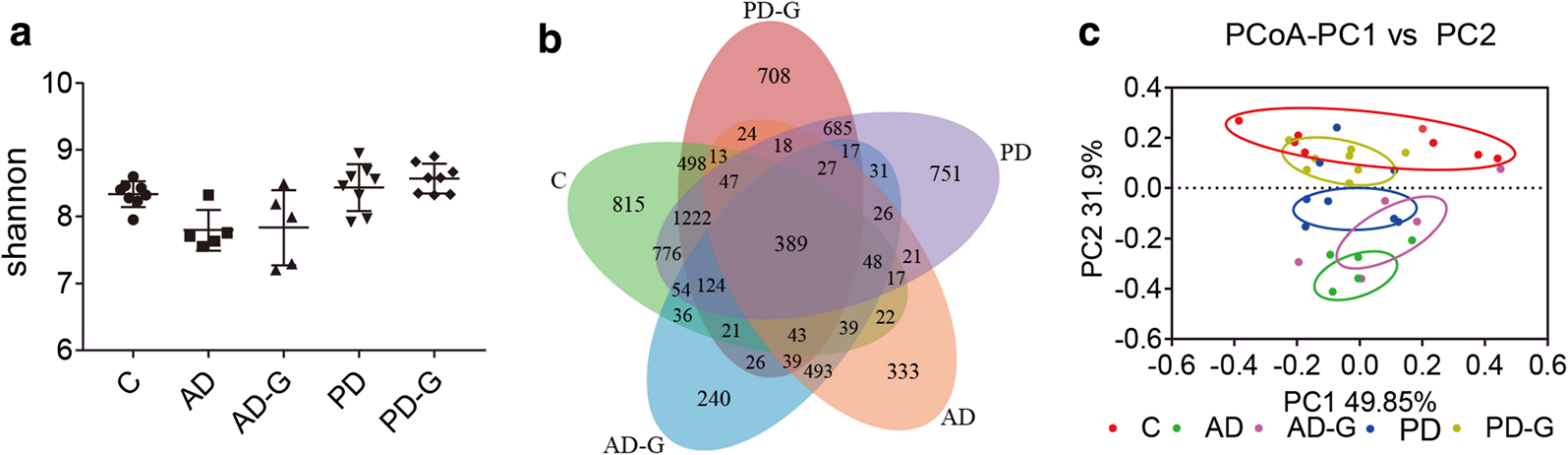
Gut microbial dysbiosis in AD and PD mice was reduced by MG1363-pMG36e-GLP-1. **a** the shannon index. **b** Petal map representation of OTUs. **c** PCoA of β diversity index. C, control group (n = 8); AD, LPS group (n = 5); AD-G, LPS + 10^9^ CFU MG1363-pMG36e-GLP-1 group (n = 5); PD, MPTP group (n = 8); PD-G, MPTP + 10^9^ CFU MG1363-pMG36e-GLP-1 group (n = 8)

**Fig. 5:**
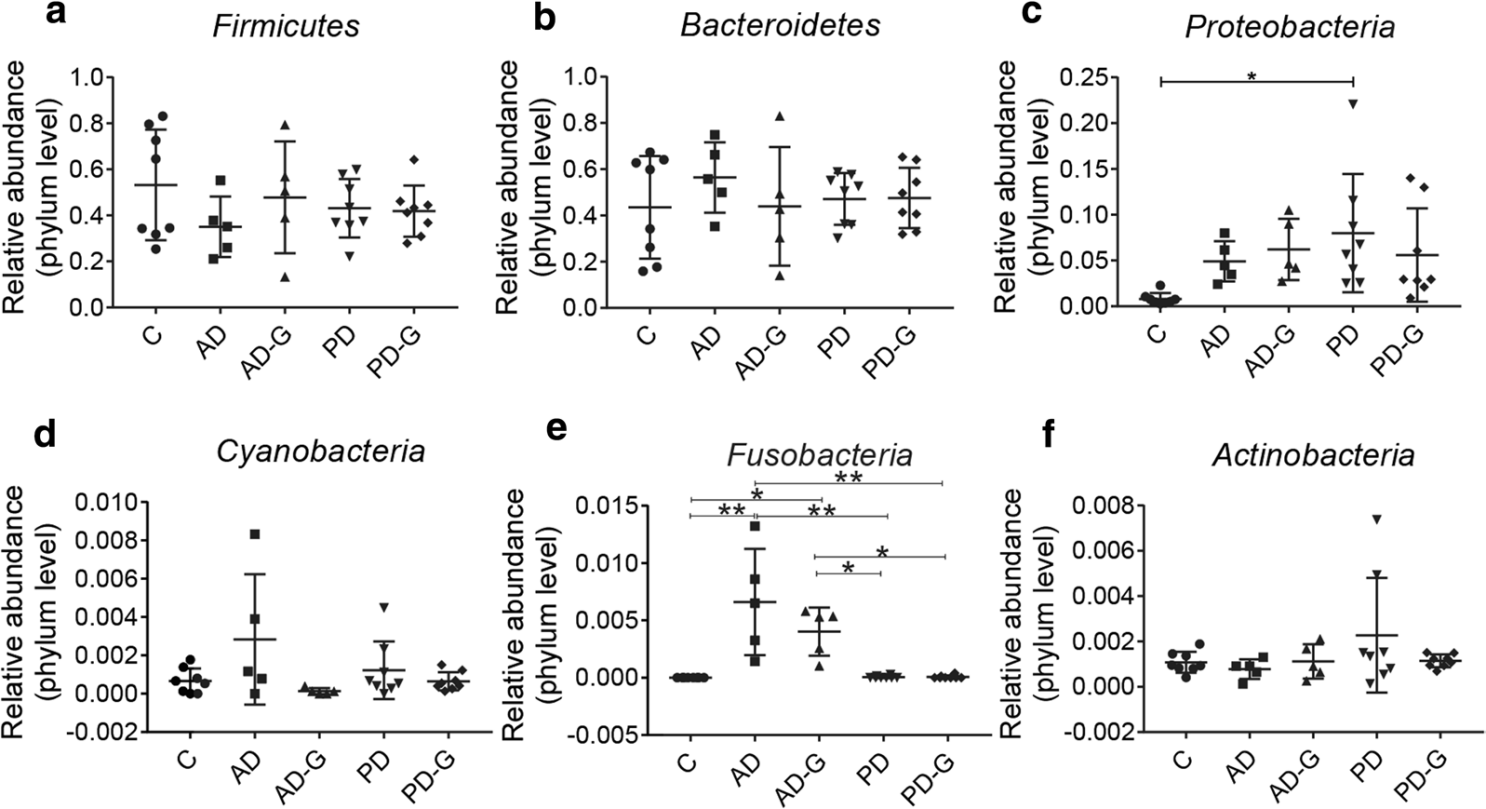
Effect of the MG1363-pMG36e-GLP-1 on the relative abundance of gut microbial diversity at phylum level in feces of mice. The relative abundance of *Firmicutes* (**a**), *Bacteroidetes* (**b**), *Proteobacteria* (**c**), *Cyanobacteria* (**d**), *Fusobacteria* (**e**), *Actinobacteria (***f**) in feces of mice.C, control group (n = 8); AD, LPS group (n = 5); AD-G, LPS + 10^9^ CFU MG1363-pMG36e-GLP-1 group (n = 5); PD, MPTP group (n = 8); PD-G, MPTP + 10^9^ CFU MG1363-pMG36e-GLP-1 group (n = 8). Data are presented as mean ± SD. *p < 0.05, **p < 0.01

**Fig. 6:**
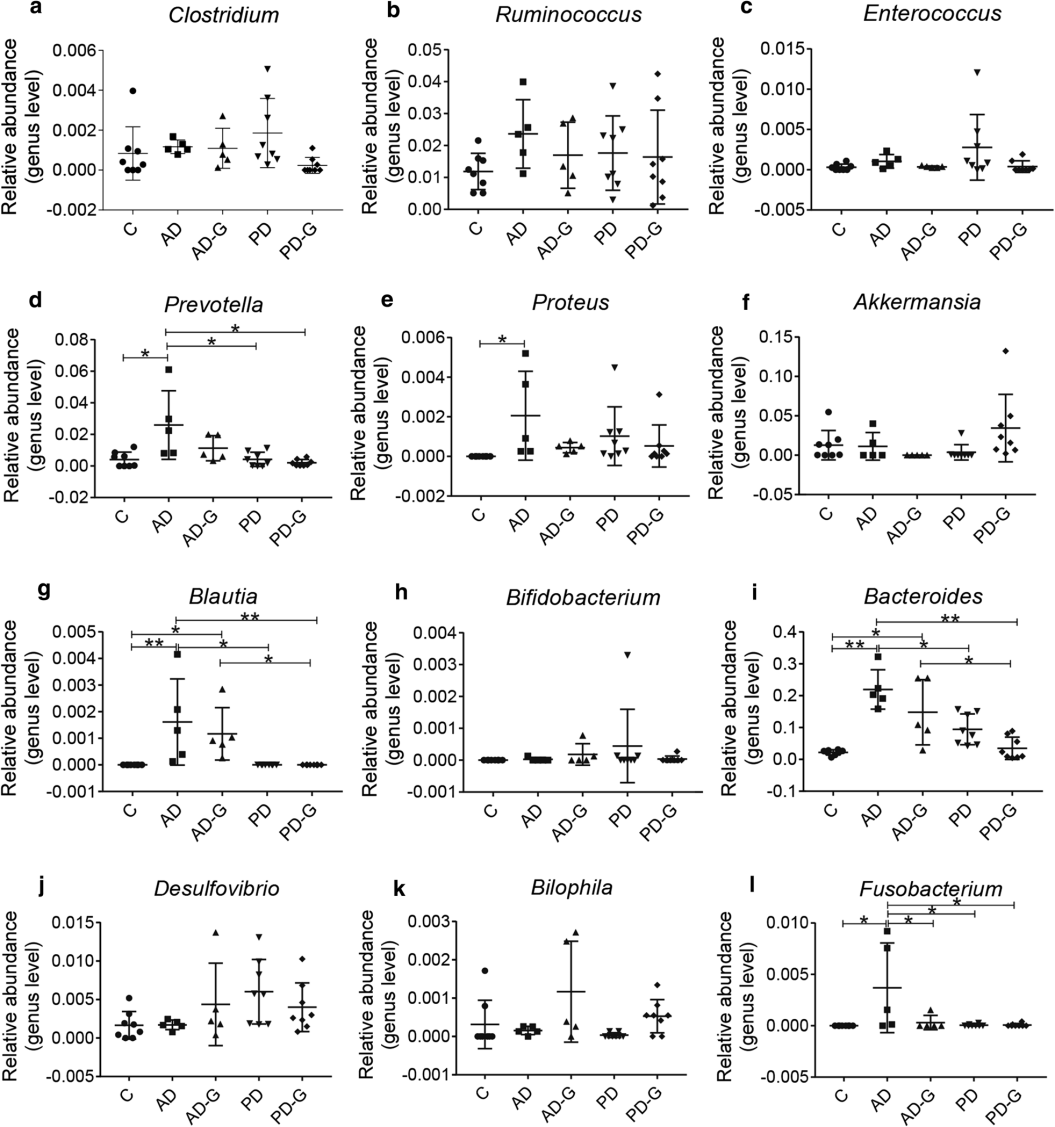
Effect of the MG1363-pMG36e-GLP-1 on the relative abundance of gut microbial diversity at genus level in feces of mice. The relative abundance of *Clostridium* (**a**), *Ruminococcus* (**b**), *Enterococcus* (**c**), *Prevotella* (**d**), *Proteus* (**e**), *Akkermansia***(f**), *Blautia* (**g**), *Bifidobacterium (***h**) *Bacteroides* (**i**) *Desulfovibrio (***j**) *Bilophila* (**k**) *Fusobacterium* (**l**) in feces of mice. C, control group (n = 8); AD, LPS group (n = 5); AD-G, LPS + 10^9^ CFU MG1363-pMG36e-GLP-1 group (n = 5); PD, MPTP group (n = 8); PD-G, MPTP + 10^9^ CFU MG1363-pMG36e-GLP-1 group (n = 8). Data are presented as mean ± SD. *p < 0.05, **p < 0.01

Injection of LPS and MPTP can cause the imbalance of intestinal microbiota in AD and PD mice, therefore high-throughput sequencing was used to evaluate the effect of MG1363-pMG36e-GLP-1 on balance of intestinal microbiota. The shannon index was used to reflect the diversity of microbial communities, which was higher in C group (8.334) and AD-G (7.836) group, compared with AD group (7.798), and the engineered strain could increase the diversity of intestinal microbial in PD group (8.434 vs. 8.569), too (Fig. 4a), but no significant difference was shown. The Venn results indicated that percent of common OTUs (389) in each group were 9.34% (389/4164, C), 24.33% (389/1599, AD), 23.53% (389/1653, AD-G), 9.15% (389/4253, PD), and 9.97% (389/3901, PD-G), respectively (Fig. 4b). The principal co-ordinates analysis (PCoA) indicated that the microbial diversity changed after the injection of LPS in AD group, while a new microbial balance was formed by the treatment of MG1363-pMG36e-GLP-1 when treated with engineered bacteria (Fig. 4c). In the end, we compared the relative abundance of probiotics and pathogens closely related to AD and PD (Figs. 5 and 6). LPS had obviously reduced the abundance of *Firmicutes* (at Phylum), *Actinobacteria* (at Phylum), *Bilophila* (at Genus), while markedly enhanced the abundance of *Bacteroidetes* (at Phylum), *Cyanobacteria* (at Phylum), *Fusobacteria* (at Phylum) (p < 0.05), *Clostridium* (at Genus), *Ruminococcus (*at Genus), *Enterococcus* (at Genus), *Prevotella* (at Genus) (p < 0.05), *Proteus* (at Genus) (p < 0.05), *Blautia* (at Genus) (p < 0.05), *Bacteroides* (at Genus) (p < 0.05) and *Fusobacterium* (at Genus) (p < 0.05) in AD group compared with C group. Taking of MG1363-pMG36e-GLP-1 (AD-G group) could reduce the pathogens of *Fusobacterium* (at Genus) (p < 0.05).

For PD mice, MPTP had obviously reduced the abundance of *Akkermansia* (at Genus), and *Bilophila* (at Genus), enhanced the number of *Proteobacteria* (at Phylum) (p < 0.05), *Cyanobacteria* (at Phylum), *Fusobacteria* (at Phylum), *Actinobacteria (*at Phylum), *Clostridium* (at Genus), *Ruminococcus* (at Genus), *Enterococcus (*at Genus), *Proteus* (at Genus), *Bacteroides* (at Genus) and *Desulfovibrio (*at Genus) in PD group compared with C group. Taking of MG1363-pMG36e-GLP-1 (PD-G group) had reduced the pathogens of *Enterococcus* (at Genus) and *Proteus* (at Genus).

## Discussion

AD and PD are diseases caused by degeneration and necrosis of neurons in the central nervous system, which seriously affect the life quality of human. However, since the current drugs used to treat AD and PD are unsatisfactory, there is an urgent need to explore their common features and develop drugs for AD and PD.

Till now, a strong connection between intestinal microbiota and nervous diseases is evidenced, and the drug GV-971 has been conditionally approved by China’s State Drug Administration for AD treatment. Although more data are needed to prove its safety and effectiveness, preclinical data supports the relationship between intestinal flora and neuroinflammation. More importantly, GV-971 treats AD by targeting to intestinal microbiota, which opens up new avenues for AD treatment by remodeling intestinal microorganisms (Wang et al. 2019). It is well known that intestinal flora imbalance occurs in the early stages of neurodegenerative diseases. The intestinal flora of NDD patients is characterized by excessive growth of pathogenic bacteria, which increases the release of LPS, and activates microglia to cause neuroinflammation (Spielman et al. 2018). In addition, disturbed intestinal flora can affect neurogenesis, development, neurotransmission and neuroimmunity (Sampson et al. 2016), which ultimately affect the pathogenesis and progress of NDD. In order to restore the disturbed intestinal microbiota to a normal level, probiotics have been widely used. Probiotics are a kind of active microorganisms that can colonize the body, remove or reduce the adhesion of pathogenic bacteria and regulate the body’s immunity, thereby protecting the host (Liu et al. 2015). Researches indicated that probiotics containing in milk could improve cognitive function in healthy elderly people (Chung et al. 2014), and could improve cognitive, sensory and emotional function in AD patients (Akbari et al. 2016). Furthermore, *L. casei* strain significantly reduced anxiety symptoms in patients with chronic fatigue syndrome (Rao et al. 2009), probiotics mixture (*L. acidophilus*, *L. casei*, and *Bifidobacterium bifidum*) reduced the overall score of the Baker Depression Scale in patients with major depression (Akkasheh et al. 2016), probiotics capsule (*L. acidophilus*, *L. casei*, *B. bifidum* and *L. fermentum*) could improve mental health and metabolic status in patients with multiple sclerosis (MS) (Kouchaki et al. 2017).

GLP-1 is an enterogenous hormone that promotes insulin secretion and reduces glucagon secretion to reduce blood glucose in a glucose concentration-dependent manner (Athauda and Foltynie 2016). Recently, epidemiology has shown that the incidence of AD and PD is much higher in patients with type 2 diabetes than that in the normal population (Marathe et al. 2013). Therefore, diabetes drugs such as GLP-1 are used to treat AD and PD, and have shown good efficacy (Aviles-Olmos et al. 2013; Gejl et al. 2016). The neuroprotective effect of GLP-1 is not only effective in AD and PD, but also in other NDD such as HD (Duarte et al. 2018), ALS (Li et al. 2012), and MS (DellaValle et al. 2016). However, as GLP-1 is easily degraded, continuous intravenous drip or continuous subcutaneous injection is required to produce therapeutic effects, which limits its clinical usage. Therefore, it is a hotspot to prevent degradation of GLP-1 as a drug (Athauda and Foltynie 2016).

In the present study, an engineered strain MG1363-pMG36e-GLP-1 was generated to continuously express GLP-1, and the LPS-induced AD model and MPTP-induced PD model were developed to evaluate the treatment effect of MG1363-pMG36e-GLP-1 on AD and PD. As we know, toll-like receptor 4 (TLR4) is a unique pattern recognition receptor (PRR), and TLR4 in the brain is mainly expressed in microglia (Seija Lehnardt et al. 2003). Toxins in the brain can specifically recognize and combine with TLR4, activate the microglia and eventually lead to neuroinflammation (Jia et al. 2017). Studies also indicate that TLR4 expression is significantly elevated in brain tissue of AD and PD patients and mice, and the aggregation of α-syn and Aβ is related to the activation of TLR4 (Seija Lehnardt et al. 2003). Similarly, our results indicated that the injection of LPS and MPTP significantly affected the behavioral ability of mice, and the oral administration of MG1363-pMG36e-GLP-1 significantly improved the behavior of mice. The immunohistochemistry results indicated that toxin injection greatly promoted the expressions of TLR4, p-IκBαand p-p65 in brain of AD and PD mice, and decreased the expression of p-AKT, p-GSK3βand β-catenin. P-AKT is a major anti-apoptotic kinase, so AKT/GSK3β signaling abnormalities are associated with many neurological diseases (Morissette et al. 2010). Previous results indicated that a lower expression of AKT in dopaminergic neurons of PD patients than that in normal people (Malagelada et al. 2008), as AKT can promote neuronal survival by mediating multiple neurotrophic factors (Morissette et al. 2010).

To further explore the connection of intestinal microbiota with AD and PD, 16S rRNA gene sequencing was used. The results indicated that LPS and MPTP had obviously changed the microbial diversity compared with the mice in control groups. In AD group, the *Firmicutes*, *Actinobacteria* showed a significant decrease at the phylum level, and the *Fusobacteria*, *Enterococcus, Proteus* showed an increase compare with control group, while MG1363-pMG36e-GLP-1 obviously reduced the pathogen *Fusobacterium* (at Genus) (p < 0.05). In PD group, MPTP obviously increased the abundance of *Proteobacteria*, *Cyanobacteria*, *Fusobacteria*, *Actinobacteria* at the phylum level, increased the abundance of *Enterococcus*, *Proteus* at the genus level and markedly decreased the abundance of *Akkermansia*, *Bilophila* at the genus level. As we know, an increase in *Fusobacterium nucleatum* (*F. nucleatum*) promotes the production of the inflammatory factors IL-8, IL-1β and TNF-α, ultimately leads to intestinal epithelial inflammation (Jia et al. 2017). *Proteus mirabilis* can cause motor dyskinesia, produce neuroinflammation and cause dopaminergic neuronal damage by producing LPS (Choi et al. 2018). Mice injected with *Nocardia astroides* (*N. astroides,* a human pathogen of the phylum *Actinobacteria*) can cause Parkinson’s like symptoms and respond to dopamine therapy, and *N. astroides* strain GUH-2 can cause apoptosis of dopamine neurons (Tam et al. 2002). For probiotics strains, *A. muciniphila* can produce secretory immunoglobulin A (IgA) and antibacterial peptides by immunological rejection to resist pathogen damage to the intestines, and has anti-inflammatory, barrier-improving properties. It can also regulate intestinal microbial balance, and improve the situation of PD, aging, pediatric autism, MS and ALS (Derrien et al. 2017; Hidalgo-Cantabrana et al. 2017).

Based on the data presented herein, we conclude that MG1363-pMG36e-GLP-1 attenuates neuroinflammation via down-regulating TLR4/NF-κB pathway, up-regulated the AKT/GSK3β signaling pathway, and restore the disturbed microbiota to normal level, so as to reduce the spatial learning and memory disorders of AD mice and the ability of movement and exploration of PD mice. Intestinal flora imbalance and neuroinflammation occur in many NDD. This study provides a strong basis for the engineering strain MG1363-pMG36e-GLP-1 in the future clinical treatment of NDD. However, we acknowledge that a control group treated AD and PD using *Lactococcus lactis* MG1363 strain will provide much more convincing evidence on the treatment of NDD for MG1363-pMG36e-GLP-1 strain.

## Data Availability

The datasets used and/or analyzed during the current study are available from the corresponding author on reasonable request.

## Code availability

Data were analyzed and graphically represented by Graphpad Prism version 7.0 (GraphPad Software, San Diego, CA, USA).
